# Clinical Characteristics and In-Hospital Outcomes of Patients Aged 90 Years or Older Admitted to an Internal Medicine Department: A Single-Center Study From Portugal

**DOI:** 10.7759/cureus.113565

**Published:** 2026-07-29

**Authors:** João Loja, Rafael Ferreira Nascimento, Maria Eduarda Moniz, Elisa Caldeira, João Miguel Freitas

**Affiliations:** 1 Internal Medicine, Serviço de Saúde da Região Autónoma da Madeira, Entidade Pública Empresarial da Região Autónoma da Madeira (SESARAM, EPERAM), Funchal, PRT; 2 Internal Medicine, Hospital Central do Funchal, Funchal, PRT

**Keywords:** aging population, geriatric medicine, hospitalization, multimorbidity, polypharmacy

## Abstract

Introduction: The number of patients aged 90 years or older requiring hospital care continues to increase. However, contemporary data describing their clinical characteristics and in-hospital outcomes in Portuguese Internal Medicine departments remain limited. This study aimed to describe the demographic characteristics, functional status, chronic medication use, primary admission diagnoses, length of hospital stay, discharge disposition, and in-hospital mortality of patients aged 90 years or older admitted to an Internal Medicine Department.

Materials and methods: A single-center retrospective descriptive study was conducted including 89 consecutive patients aged 90 years or older admitted to the Internal Medicine Department of a Portuguese tertiary hospital between January and December 2025. Functional status was assessed using the Barthel Index, and comorbidity was evaluated with the Charlson Comorbidity Index.

Results: Eighty patients (90%) were nonagenarians, and nine (10%) were centenarians. Most patients were female (n = 57, 64%), with a mean age of 94 ± 3.2 years. Before admission, 69 (77%) lived at home, whereas 20 (23%) were institutionalized. At admission, 48 (54%) patients were completely dependent, 30 (34%) were partially dependent, and 11 (12%) were independent in activities of daily living. Almost all patients (n = 88, 99%) were receiving chronic medication, and 54 (61%) met the criteria for polypharmacy (≥5 medications). Antihypertensive therapy was prescribed in 61 (69%) patients. Among the 32 patients treated with antihypertensive monotherapy, an angiotensin-converting enzyme inhibitor was the most prescribed drug (n = 16, 50%). Antipsychotics were prescribed in 29 (33%) patients and benzodiazepines in 24 (27%). Respiratory tract infection was the most frequent admission diagnosis (n = 47, 53%), followed by urinary tract infection (n = 22, 25%) and hyponatremia (n = 6, 7%). The mean hospital stay was 10 ± 7.8 days (range, 2-46 days). Overall, 74 (83%) patients were discharged, whereas 15 (17%) died during hospitalization. All patients who died were completely dependent at admission.

Conclusions: Patients aged 90 years or older admitted to an Internal Medicine Department commonly presented with functional dependence, multiple comorbidities, and polypharmacy. Respiratory tract infections were the leading cause of hospitalization, and in-hospital mortality was 17%. These findings provide contemporary data on the characteristics and hospital outcomes of very old adults and support the importance of comprehensive clinical assessment, including regular review of chronic medication, in this growing patient population.

## Introduction

Population aging has become one of the most significant demographic and public health challenges worldwide [1]. Advances in healthcare, living conditions, and socioeconomic development have contributed to a sustained increase in life expectancy, resulting in a growing number of adults aged 90 years or older [1]. In Portugal, life expectancy at birth now exceeds 80 years, and this age group represents an increasing proportion of the population, mirroring demographic trends observed across Europe [2].

The rising number of very old adults has been accompanied by an increase in hospital admissions. Patients aged 90 years or older frequently present with multiple chronic conditions, functional dependence, and polypharmacy, factors that increase the complexity of inpatient care and are associated with prolonged hospitalization, higher healthcare utilization, and increased in-hospital mortality [3,4]. Respiratory infections, cardiovascular diseases, and metabolic disorders remain among the most common reasons for admission in this population [4,5].

Although several studies have described the clinical characteristics and outcomes of hospitalized patients aged 90 years or older, most evidence originates from heterogeneous healthcare settings or nationwide administrative databases [3-6]. Contemporary data from Portuguese Internal Medicine departments remain limited, despite these departments providing care for a large proportion of older adults with complex multimorbidity. Furthermore, information describing the demographic profile, functional status, chronic medication use, admission diagnoses, and hospital outcomes of this population in Portugal is scarce.

Chronic medication use represents an important aspect of the clinical assessment of very old adults because polypharmacy is highly prevalent and may influence functional status, adverse drug events, and clinical outcomes. In this study, particular attention was given to antihypertensive agents, antipsychotics, and benzodiazepines, as these are among the most frequently prescribed medication classes in older adults and contribute substantially to the overall medication burden in this population. Therefore, this study aimed to describe the demographic characteristics, functional status, chronic medication use, primary admission diagnoses, length of hospital stay, discharge disposition, and in-hospital mortality of patients aged 90 years or older admitted to a Portuguese Internal Medicine Department during 2025.

## Materials and methods

Study design and setting

A single-center retrospective descriptive cohort study was conducted at the Internal Medicine Department of the Central Hospital of Funchal, Madeira, Portugal, a tertiary referral hospital providing medical care to the population of the Autonomous Region of Madeira. The study included patients aged 90 years or older admitted between January 1 and December 31, 2025.

Study population

All consecutive unique patients aged 90 years or older admitted to the Internal Medicine Department between January 1 and December 31, 2025, were eligible for inclusion. A total of 89 patients met the inclusion criteria and were included in the final analysis. Each patient was included only once, and repeated hospital admissions during the study period were not considered. No patients were excluded because of incomplete medical records or missing data. Patients transferred from other hospital departments, receiving palliative care, or readmitted during the study period were not included in the study.

Data collection

Clinical data were obtained through a retrospective review of electronic medical records and hospital discharge documentation. The following variables were collected: age; sex; place of residence before admission (home or long-term care facility); functional status at hospital admission; chronic medication use before admission; use of antihypertensive drugs, antipsychotics, and benzodiazepines; primary admission diagnosis; length of hospital stay; discharge disposition; and in-hospital mortality.

The primary admission diagnosis was defined according to the admitting physician's diagnosis. Length of hospital stay was calculated as the number of days between hospital admission and discharge or in-hospital death.

Functional status assessment

Functional status at hospital admission was assessed using the Barthel Index [7], as routinely documented in the medical records. Patients were classified as independent (Barthel Index 91-100), partially dependent (21-90), or totally dependent (0-20). The Barthel Index reflected the patient's functional status prior to the acute illness that led to hospitalization, and it was retrospectively extracted from medical records. Barthel Index data were available for all included patients; therefore, no missing data handling was required.

Medication assessment

Chronic medication use was defined as regularly prescribed medications documented before hospital admission. Vitamin supplements, topical agents, and medications prescribed on an as-needed basis were excluded.

Polypharmacy was defined as the chronic use of five or more medications. Antihypertensive therapy was classified as monotherapy when a single antihypertensive agent was prescribed and as combination therapy when two or more antihypertensive agents were prescribed. Antihypertensive drug classes included angiotensin-converting enzyme inhibitors, angiotensin receptor blockers, beta-blockers, calcium channel blockers, diuretics, and other antihypertensive agents.

The use of antipsychotics and benzodiazepines referred to chronic treatment before hospital admission. Benzodiazepines were classified based on their plasma half-life into intermediate-acting (12 to 24 hours) and long-acting (>24 hours).

Statistical analysis

This was a descriptive study. Continuous variables are presented as mean ± standard deviation (SD) or median and interquartile range (IQR), according to their distribution. Categorical variables are reported as absolute frequencies and percentages. Descriptive statistical analyses were performed using Microsoft Excel for Microsoft 365 (Microsoft Corporation, Redmond, WA, USA). Percentages were calculated using the number of patients with available data for each variable.

Ethical considerations

The ethics committee of Serviço de Saúde da Região Autónoma da Madeira (SESARAM) approved the current study in accordance with the Helsinki Declaration statements. The requirement for informed consent was waived because no intervention was performed and all data were analyzed anonymously in accordance with institutional policies and applicable data protection regulations.

## Results

A total of 89 unique patients aged 90 years or older were included in the study. Of these, 80 (90%) were aged 90-99 years and nine (10%) were aged 100 years or older. The mean age was 93 ± 2.3 years for nonagenarians and 101 ± 1.8 years for centenarians. Most patients were female (57, 64%). Before hospitalization, 69 patients (77%) lived at home, whereas 20 (23%) resided in long-term care facilities.

At hospital admission, 48 patients (54%) were completely dependent for activities of daily living, 30 (34%) were partially dependent, and 11 (12%) were independent according to the Barthel Index. Eighty-eight patients (99%) were receiving at least one chronic medication before admission. Polypharmacy was present in 54 patients (61%). Among patients with polypharmacy, 25 (46%) were completely dependent for activities of daily living.

Baseline demographic and clinical characteristics are summarized in Table 1.

**Table 1:** Baseline characteristics of nonagenarians and centenarians admitted to Internal Medicine wards Upon admission to the wards, patients were classified according to their degree of dependence in activities of daily living based on their BI scores: independent if the score was 91-100, partially dependent if the score was 21-90, and totally dependent if the score was 0-20. Polypharmacy was defined as the chronic concomitant use of five or more medications prior to hospitalization. SD: standard deviation; BI: Barthel Index

	Nonagenarians (n = 80)	Centenarians (n = 9)
Age, years, mean ± SD	93 ± 2.3	101 ± 1.8
Gender, n (%)		
Male	49 (55%)	8 (9%)
Female	31 (35%)	1 (1%)
Living at home, n (%)	64 (72%)	5 (6%)
Institutionalized, n (%)	16 (18%)	4 (4%)
BI category, n (%)		
Totally dependent in daily life activities	41 (46%)	7 (8%)
Partially dependent in daily life activities	28 (31%)	2 (2%)
Independent in daily life activities	11 (12%)	0 (0%)
Polypharmacy, n (%)	52 (58%)	2 (2%)

Chronic antihypertensive therapy was prescribed in 61 patients (69%). Of these, 32 (52%) received monotherapy, and 29 (48%) received combination therapy. Angiotensin-converting enzyme inhibitors were the most frequently prescribed agents used as monotherapy, whereas an angiotensin receptor blocker combined with a thiazide diuretic was the most common combination regimen (Table 2).

**Table 2:** Chronic antihypertensive therapy of patients admitted to Internal Medicine wards ACEI: angiotensin-converting enzyme inhibitor; ARB: angiotensin II receptor blocker; CCB: calcium channel blocker

Chronic antihypertensive therapy (n = 61)	
Monotherapy, n	32
ACEI, n (%)	16 (50%)
ARB, n (%)	9 (28%)
CCB, n (%)	7 (22%)
Combination therapy, n	29
ARB + thiazide diuretic, n (%)	11 (38%)
ARB + CCB, n (%)	8 (28%)
ACEI + CCB, n (%)	6 (21%)
ACEI + thiazide diuretic, n (%)	3 (10%)
ACEI + CCB + thiazide diuretic, n (%)	2 (7%)

Antipsychotic medications were prescribed in 29 patients (33%), whereas chronic benzodiazepine therapy was documented in 24 patients (27%). Among benzodiazepine users, 13 (54%) received an intermediate-acting agent and 11 (46%) a long-acting agent (Table 3).

**Table 3:** Distribution of benzodiazepines used chronically by patients admitted to Internal Medicine wards Benzodiazepines were classified based on their plasma half-life into intermediate-acting (12 to 24 hours) and long-acting (>24 hours).

Benzodiazepines used chronically (n = 24)	
Intermediate-acting benzodiazepines, n	13
Alprazolam, n (%)	9 (69%)
Lorazepam, n (%)	4 (31%)
Long-acting benzodiazepines, n	11
Diazepam, n (%)	5 (45%)
Clonazepam, n (%)	3 (27%)
Cloxazolam, n (%)	2 (18%)
Mexazolam, n (%)	1 (9%)

Respiratory tract infection was the most frequent primary admission diagnosis, accounting for 50 patients (56%), followed by urinary tract infection (n = 22, 25%) and hyponatremia (n = 6,7%). The complete distribution of primary admission diagnoses is presented in Table 4.

**Table 4:** Admission diagnosis of patients hospitalized in Internal Medicine wards

Admission diagnosis	
Diseases of the respiratory system	
Community-acquired pneumonia, n (%)	24 (27%)
Nosocomial pneumonia, n (%)	15 (17%)
Acute tracheobronchitis, n (%)	6 (7%)
Influenza A flu, n (%)	5 (6%)
Pleural effusion, n (%)	1 (1%)
Diseases of the genitourinary system	
Acute pyelonephritis, n (%)	13 (15%)
Acute cystitis, n (%)	9 (10%)
Endocrine, nutritional, and metabolic diseases	
Hyponatremia, n (%)	6 (7%)
Diseases of the nervous system	
Ischemic stroke, n (%)	4 (4%)
Diseases of the circulatory system	
Acute pulmonary edema	2 (2%)
Infectious and parasitic diseases	
Acute gastroenteritis	1 (1%)
Erysipelas	1 (1%)
Diseases of the digestive system	
Acute pancreatitis	1 (1%)
Upper gastrointestinal bleeding	1 (1%)

The median length of hospital stay was eight days (IQR, 6-12 days; range, 2-46 days). Among survivors, the median length of stay was eight days (IQR, 6-11 days), whereas among non-survivors, it was 11 days (IQR, 8-18 days), as seen in Table 5.

**Table 5:** Distribution of surviving and non-surviving patients according to length of stay IQR: interquartile range

Length of stay (in days)			
	Median	IQR	Range
Survivors	8	6-11	2-46
Non-survivors	11	8-18	3-34

Overall, 74 patients (83%) were discharged alive, and 15 (17%) died during hospitalization. Sixty-two patients (84% of survivors) were discharged to their home and 12 (16%) to a long-term care facility (Table 6).

**Table 6:** Discharge disposition

Discharge disposition (n = 74)	
Discharge to home, n (%)	62 (84%)
Discharge to long-term care facility, n (%)	12 (16%)

All 15 non-survivors were completely dependent for activities of daily living at hospital admission, whereas no deaths occurred among patients who were partially dependent or independent. Among the non-survivors, 10 (67%) were female, eight (54%) resided in long-term care facilities before admission, and polypharmacy was found in six (40%) patients (Table 7).

**Table 7:** Distribution of surviving and non-surviving patients according to gender, place of origin prior to admission, level of dependency prior to admission, and presence or absence of polypharmacy Upon admission to the wards, patients were classified according to their degree of dependence in activities of daily living based on their BI scores: independent if the score was 91-100, partially dependent if the score was 21-90, and totally dependent if the score was 0-20. Polypharmacy was defined as the chronic concomitant use of five or more medications prior to hospitalization. BI: Barthel Index

	Survivors (n = 74)	Non-survivors (n = 15)
Gender, n (%)		
Male	27 (36%)	5 (33%)
Female	47 (64%)	10 (67%)
Living at home, n (%)	62 (84%)	7 (46%)
Institutionalized, n (%)	12 (16%)	8 (54%)
BI category, n (%)		
Totally dependent in daily life activities	34 (46%)	15 (100%)
Partially dependent in daily life activities	29 (39%)	0 (0%)
Independent in daily life activities	11 (15%)	0 (0%)
Polypharmacy, n (%)	48 (65%)	6 (40%)

## Discussion

This study provides a contemporary descriptive overview of patients aged 90 years or older admitted to an Internal Medicine Department in Portugal. Most patients were female and had a high prevalence of functional dependence and polypharmacy. Respiratory tract infection was the most frequent primary admission diagnosis, and the in-hospital mortality rate was 17%.

The predominance of female patients is consistent with previous studies of hospitalized nonagenarians, in which women accounted for approximately two-thirds of the study population. This finding likely reflects the longer life expectancy of women and the demographic structure of the oldest-old population in Portugal and other European countries, where women constitute the majority of individuals aged 90 years or older [8]. As population aging continues, the number of very old adults requiring hospital care is expected to increase, posing important challenges for Internal Medicine services [9].

More than half of the patients were completely dependent for activities of daily living at hospital admission. In addition, all patients who died during hospitalization were completely dependent. Although this descriptive finding may be clinically relevant, the present study was not designed to determine whether functional dependence independently predicts mortality. Differences in illness severity, treatment limitations, comorbidity burden, or other unmeasured factors may have contributed to this observation. Nevertheless, previous prospective studies have reported an association between impaired functional status and adverse outcomes among hospitalized nonagenarians [10]. These findings support the importance of assessing functional status as part of the comprehensive clinical evaluation of very old adults admitted to hospital [11].

Polypharmacy was identified in 61% of the study population, reflecting the substantial chronic medication burden in this age group. Antihypertensive agents, antipsychotics, and benzodiazepines were among the most frequently prescribed medication classes. Previous studies have shown that polypharmacy and the use of psychotropic medications are common in older adults and have been associated with adverse outcomes, including falls, delirium, and functional decline [12,13]. However, these outcomes were not evaluated in the present study, and medication appropriateness was not assessed using validated instruments such as the STOPP/START criteria or the Beers Criteria. Consequently, our findings should not be interpreted as evidence of inappropriate prescribing or medication-related harm. Instead, they highlight the frequency of chronic medication use in this population and support the importance of regular medication review as part of comprehensive geriatric care.

Interestingly, polypharmacy was less frequent among non-survivors than in the overall cohort. Given the descriptive nature of the study and the small number of deaths, no conclusions can be drawn regarding this finding. Possible explanations include treatment de-escalation in patients with advanced illness, differences in prescribing practices, incomplete medication documentation, or random variation.

Respiratory tract infections were the leading cause of hospital admission, followed by urinary tract infections and hyponatremia. These findings are consistent with previous reports identifying respiratory infections as one of the most common reasons for hospitalization among very old adults [14,15]. However, the present study did not evaluate infection severity, microbiological findings, aspiration risk, vaccination status, or infection-related mortality. Therefore, comparisons with studies investigating these factors should be interpreted with caution.

The mean length of hospital stay was 10 days, comparable to that reported in previous observational studies of hospitalized nonagenarians. The observed in-hospital mortality of 17% was also within the range reported in the literature [4]. Nevertheless, direct comparisons between studies should be made cautiously because differences in study design, healthcare setting, patient selection, admission diagnoses, and case mix may substantially influence mortality rates.

This study has several limitations. Its retrospective design depended on information available in medical records and may therefore be subject to incomplete documentation or misclassification of some variables. The study included a relatively small number of patients from a single tertiary hospital, including only nine centenarians, which limits the generalizability of the findings. In addition, the absence of a comparison group precludes assessment of factors associated with hospital outcomes, and the descriptive design does not permit inference regarding predictors or causal relationships. Functional status was assessed at hospital admission and may have been influenced by the acute illness. Measures of frailty, cognitive status, delirium, illness severity, palliative-care status, medication appropriateness, adverse drug events, and post-discharge outcomes were not available. Finally, the study reflects the experience of a single Internal Medicine Department and may not be representative of other hospitals or healthcare systems.

Despite these limitations, this study provides contemporary descriptive data on patients aged 90 years or older admitted to a Portuguese Internal Medicine Department, a population for which published national data remain limited. These findings contribute to the characterization of this growing patient population and may help inform future multicenter studies designed to evaluate clinical outcomes and optimize hospital care.

## Conclusions

In this single-center retrospective descriptive study, patients aged 90 years or older admitted to an Internal Medicine Department commonly had a high prevalence of functional dependence and polypharmacy, while respiratory tract infection was the most frequent primary admission diagnosis. In-hospital mortality was 17%, and all non-survivors were completely dependent at admission; however, the descriptive study design does not allow identification of independent predictors of mortality.

As the population of adults aged 90 years or older continues to increase, further multicenter studies are needed to better characterize this population and evaluate factors such as functional status, illness severity, medication appropriateness, and post-discharge outcomes. Such evidence may help inform healthcare planning and optimize the care of this growing patient population.
